# Transcriptome Sequencing and Developmental Regulation of Gene Expression in *Anopheles aquasalis*

**DOI:** 10.1371/journal.pntd.0003005

**Published:** 2014-07-17

**Authors:** André L. Costa-da-Silva, Osvaldo Marinotti, José M. C. Ribeiro, Maria C. P. Silva, Adriana R. Lopes, Michele S. Barros, Anderson Sá-Nunes, Bianca B. Kojin, Eneas Carvalho, Lincoln Suesdek, Mário Alberto C. Silva-Neto, Anthony A. James, Margareth L. Capurro

**Affiliations:** 1 Laboratório de Mosquitos Geneticamente Modificados, Departamento de Parasitologia, Instituto de Ciências Biomédicas, Universidade de São Paulo, São Paulo, São Paulo, Brazil; 2 Instituto Nacional de Ciência e Tecnologia em Entomologia Molecular, INCT-EM, Rio de Janeiro, Rio de Janeiro, Brazil; 3 Department of Molecular Biology and Biochemistry, University of California Irvine, Irvine, California, United States of America; 4 Section of Vector Biology, Laboratory of Malaria and Vector Research, National Institute of Allergy and Infectious Diseases, National Institutes of Health, Bethesda, Maryland, United States of America; 5 Laboratório de Bioquímica e Biofísica, Instituto Butantan, São Paulo, São Paulo, Brazil; 6 Laboratório de Imunologia Experimental, Departamento de Imunologia, Instituto de Ciências Biomédicas, Universidade de São Paulo, São Paulo, São Paulo, Brazil; 7 Centro de Biotecnologia, Instituto Butantan, São Paulo, São Paulo, Brazil; 8 Laboratório de Parasitologia, Instituto Butantan, São Paulo, São Paulo, Brazil; 9 Laboratório de Sinalização Celular, Instituto de Bioquímica Médica, Universidade Federal do Rio de Janeiro, Rio de Janeiro, Rio de Janeiro, Brazil; 10 Department of Microbiology and Molecular Genetics, University of California Irvine, Irvine, California, United States of America; Johns Hopkins Bloomberg School of Public Health, United States of America

## Abstract

**Background:**

*Anopheles aquasalis* is a major malaria vector in coastal areas of South and Central America where it breeds preferentially in brackish water. This species is very susceptible to *Plasmodium vivax* and it has been already incriminated as responsible vector in malaria outbreaks. There has been no high-throughput investigation into the sequencing of *An. aquasalis* genes, transcripts and proteins despite its epidemiological relevance. Here we describe the sequencing, assembly and annotation of the *An. aquasalis* transcriptome.

**Methodology/Principal Findings:**

A total of 419 thousand cDNA sequence reads, encompassing 164 million nucleotides, were assembled in 7544 contigs of ≥2 sequences, and 1999 singletons. The majority of the *An. aquasalis* transcripts encode proteins with their closest counterparts in another neotropical malaria vector, *An. darlingi*. Several analyses in different protein databases were used to annotate and predict the putative functions of the deduced *An. aquasalis* proteins. Larval and adult-specific transcripts were represented by 121 and 424 contig sequences, respectively. Fifty-one transcripts were only detected in blood-fed females. The data also reveal a list of transcripts up- or down-regulated in adult females after a blood meal. Transcripts associated with immunity, signaling networks and blood feeding and digestion are discussed.

**Conclusions/Significance:**

This study represents the first large-scale effort to sequence the transcriptome of *An. aquasalis*. It provides valuable information that will facilitate studies on the biology of this species and may lead to novel strategies to reduce malaria transmission on the South American continent. The *An. aquasalis* transcriptome is accessible at http://exon.niaid.nih.gov/transcriptome/An_aquasalis/Anaquexcel.xlsx.

## Author Summary

The mosquito *Anopheles aquasalis* is responsible for transmitting malaria parasites to humans in South America coastal areas. *An. aquasalis* females transmit *Plasmodium vivax* and *Plasmodium falciparum*, the two major malaria etiological agents in these endemic sites. Although the vectorial importance of this mosquito has been demonstrated, molecular aspects of its biology have been poorly explored. In this study, we present the transcriptome of *An. aquasalis* using 454 sequencing followed by automated bioinformatic analyses. Our study identified and annotated more than 9,000 putative proteins based on homology, gene ontology, and/or biochemical pathways, including putative secretory proteins. The comparison of RNAs present in samples extracted from larvae, sugar fed adult females, or blood fed adult females, reveal gene expression regulation during mosquito development. The present dataset provides a useful resource and adds greatly to our understanding of a human malaria vector from developing countries.

## Introduction

*Anopheles aquasalis* is a neotropical malaria vector, found along the northern coast of South America. Its geographical distribution extends from Brazil to Panama on the Atlantic shore and from Panama to Ecuador along the Pacific coast [1]–[6]. Their larvae develop preferentially in brackish water such as in mangrove swamps and coastal ground pools, but they also are capable of living in fresh water and often occur several kilometers from the coast [7]. The species has been reported as malaria vector in Venezuela, Brazil, Trinidad and The Caribbean [8]–[12]. *Plasmodium falciparum* and *P. vivax*, the two main human malaria parasites, are transmitted by *An. aquasalis* [12], [13]. Its epidemiological importance was confirmed during a *P. vivax* malarial outbreak in 1991–1992 occurred in Trinidad which was linked to *An. aquasalis* [14].

Despite its importance as a malaria vector in Central and South America, regions responsible for 22% of the global area at risk of *P. vivax* transmission [15], little is known about its genome, transcriptome and proteome. Efforts to colonize this species [12], [16] provided the basis for studies assessing development, gene expression and immune responses to *Plasmodium* infection [12], [17]–[24]. The role of reactive oxygen species and JAK-STAT pathway in the control of *P. vivax* infection were characterized in *An. aquasalis*. However, it was also demonstrated that this anopheline promotes an apparent weak response against *P. vivax* infection [18] which is supposed to be related to its susceptibility to this parasite species. Corroborating with this hypothesis, a recent work showed that colonized *An. aquasalis* presents higher infection rates and oocyst numbers when compared to other Neotropical anophelines [25].

Here we describe the transcriptomes and deduced proteomes of *An. aquasalis* larvae and adults fed on sugar and on blood. This data set provides indispensable information for the systematic and comprehensive analysis of molecules that may play an active role in mosquito biology and malaria transmission.

## Methods

### Mosquitoes

The *An. aquasalis* colony (gift from Dr. Paulo Filemon Paolucci Pimenta/Fiocruz) was maintained in the insectary at the Departamento de Parasitologia, ICB-USP (São Paulo, Brazil) at 27±1°C, 75–80% relative humidity and 12 h light∶12 h dark photoperiod. Larvae were kept in 0.2% marine salt (w/v), and were fed with powdered fish food (Tetramin, Blacksburg, VA, USA). Adult males and females were kept together in a cage with access to a 10% sucrose solution (w/v) *ad libitum*. Female mosquitoes aged 5–7 days after emerging from the pupal stage were allowed to feed on anaesthetized mice for 30 minutes. Eggs were collected 2–3 days post blood meal and hatched in 0.2% marine salt (w/v).

### RNA extraction and quantification

Larvae RNA was extracted from a pool of third and fourth instar larvae (20 each). RNA also was extracted from one pool of twenty 5–7 day old adults females fed with sucrose and two additional samples, each composed of twenty 5–7 day old adult females at 24 h after blood meal. Frozen animals were used for mRNA extraction using magnetic beads covalently bound to oligo(dT) tags (Dynabeads mRNA DIRECT, Invitrogen, Grand Island, NY, USA), accordingly to the manufacturer's instructions. Aliquots of the purified mRNA samples were quantified using Quant-iTRiboGreen RNA Reagent (Invitrogen) and their integrity was checked in a microfluidics-based platform (Agilent 2100 Bioanalyzer, Santa Clara, CA, USA).

### Sequencing

Approximately 400 ng of polyA+ RNA from each sample were used as template for sequencing. mRNA was fragmented with zinc chloride, resulting in molecules with a size distribution range from 300 to 800 bases (assessed by the Bioanalyzer), and used as a template for cDNA synthesis. Adaptors were linked to the fragment ends. Beads coated with oligonucleotides complementary to the adaptor sequences were incubated with the cDNA fragments, and a water-in-oil emulsion was produced, followed by emulsion PCR. Washed beads were deposited in picotiter plate wells, and other sequencing reagents were loaded on the 454 GS-Junior sequencer (Roche, Branford, CT, USA). Two hundred sequencing cycles were performed. Base-calling was performed by the 454 GS-Junior data processing software GS Run Processor, version 2.7.

### Assembly and annotation

The blastn tool (performed locally from executables obtained at the NCBI FTP site ftp://ftp.ncbi.nih.gov/blast/executables/) [26] and CAP3 assembler [27] were used for sequence clustering, by a decreasing word size inclusion strategy as described in detail previously [28]. Coding sequences (CDS) were extracted as described before [28] based on matches to public databases or longer open reading frames with a signal peptide indicative of secretion. Contigs are named Anoaqua-XXX or megaclu_asbSigP-XXX reflecting the two modes of data extraction, where XXX represents the number of the full length assembled contig. Reference to specific contigs in this will use an abbreviated notation, contigXXX, instead of the full CDS name. The data was organized in a hyperlinked spreadsheet (Anaquexcel) as described in [29]. The blastx [30] tool was used to compare the translated nucleotide sequences to the NR protein database of the NCBI and to the Gene Ontology (GO) database [31]. The tool, reverse position-specific BLAST (rpsblast) [30], was used to search for conserved protein domains in the Pfam [32], SMART [33], KOG [34] and conserved domains databases (CDD) [35]. Predicted protein segments starting with a methionine were submitted to the SignalP server [36] to identify translation products that could be secreted. Glycosylation sites on the proteins were predicted with the program NetOGlyc [37]. Functional annotation of the transcripts was based on all of the comparisons above. Transcripts and their encoded proteins were classified based on function and/or protein families. To compare gene expression between libraries, paired comparisons of the number of reads hitting each contig were calculated by *Χ* ^2^ tests to detect significant differences between samples when the minimum expected value was larger than 5 and P<0.05. A 2-fold change (up or down) was considered of interest when statistically significant. Normalized fold-ratios of the library reads were computed by adjusting the numerator by a factor based on the ratio of the total number of reads in each library, and adding one to the denominator to avoid division by zero. The complete Anaquexcel dataset (including links) may be downloaded from http://exon.niaid.nih.gov/transcriptome/An_aquasalis/Anaquexcel.xlsx and searched as an Excel spreadsheet. The raw data were deposited to the Sequence Read Archives (SRA) of the National Center for Biotechnology Information (NCBI) under bioproject number PRJNA210899, biosamples SRS455914 (adults) and SRS455922 (larvae) and runs SRR927455 (female sucrose), SRR927456 (female blood fed) and SRR927458 (L3+L4 larvae). CDS representing >90% of known proteins or larger than 250 amino acids were deposited to the Transcriptome Shotgun Annotation (TSA) portal of the NCBI and received the accession numbers from GAMD01000001 to GAMD01003464.

### Validation of RNA-seq data by qRT-PCR

To confirm the expression profile generated by the transcriptome sequencing, we validated the expression levels of twelve contigs identified in RNA-seq analysis using a qRT-PCR method. Contigs classified as enhanced or specific for larva (Anoaqua-397, Anoaqua-1598, Anoaqua-4095, Anoaqua-17360 and Anoaqua-1222), specific for adult (Anoaqua-436 and Anoaqua-457) and blood meal regulated (megaclu_asbSigP-9948, Anoaqua-3237, megaclu_asbSigP-2537, Anoaqua-5059 and Anoaqua-24500) were analyzed.

To perform these quantifications, TRIZOL reagent (Invitrogen) was used to extract total RNA from 3 independent biological pools of third and fourth instar larvae (10 each), ten 5–7 day old adults females fed with sucrose and ten 5–7 day old adult females at 24 h after blood meal. For each extraction, total RNA was quantified and 4.0 µg were treated with DNAse I (Invitrogen) and was reverse transcribed using superscript II (Invitrogen) and oligoDT (Invitrogen) in a 40 µL final reaction volume.

qRT-PCR assay was performed in Mastercycler Realplex 2 thermocycler (Eppendorf) with Maxima SYBR green Master Mix (Thermo Scientific). Reactions were performed in a 20 µL final volume containing 2 µL of cDNA template and 0.5 µM of each primer (Table S3). Primers pair amplification efficiency was estimated using original cDNA in seven-fold serial dilutions to generate a standard curve. All primers pair showed efficiency greater than 90% (Table S3) and only one specific peak was observed in the melting curve for each analyzed transcript. Each sample was measured in triplicate and three biological replicates were quantified. Expression levels of *An. aquasalis* Rp49 constitutive gene [18], [38] (Tables S3 and S4) was used to normalize variation in total cDNA concentration as an endogenous control. Fold-changes in gene expression were estimated by delta-delta C_T_ method [39] and sample with lower expression levels for each gene was defined as calibrators.

### Statistical analysis

Statistical significance was evaluated using Graph Pad Prism5 software. Data was checked in relation to normality using D'Agostino and Pearson omnibus normality test. One way ANOVA followed by Tukey's Multiple Comparison posttest were applied when the data adequate to parametric model. Non-parametric data was analyzed by Kruskal-Wallis test followed by Dunn's Multiple Comparison posttest. Confidence intervals of 95% were defined.

## Results/Discussion

### General description of the *An. aquasalis* transcriptome

Sequencing returned 1.1–1.7×10^5^ reads among the samples classes, with averages sizes ranging from 350–420 nucleotides in length and 48–60×10^6^ total bases sequenced (Table 1). Approximately 7% of these were ribosomal RNAs and therefore were excluded in the subsequent analyses.

**Table 1:** *Anopheles aquasalis* sequencing results.

Sample	No. reads	average size (nt)	total sequenced bases	% rRNA
sucrose-fed adult female	131,678	418	55,152,581	7.4
blood-fed adult female^1^	172,244	350	60,423,895	7.0
larvae	115,771	420	48,674,928	7.2

1 Includes all blood-fed samples.

**Figure 1:**
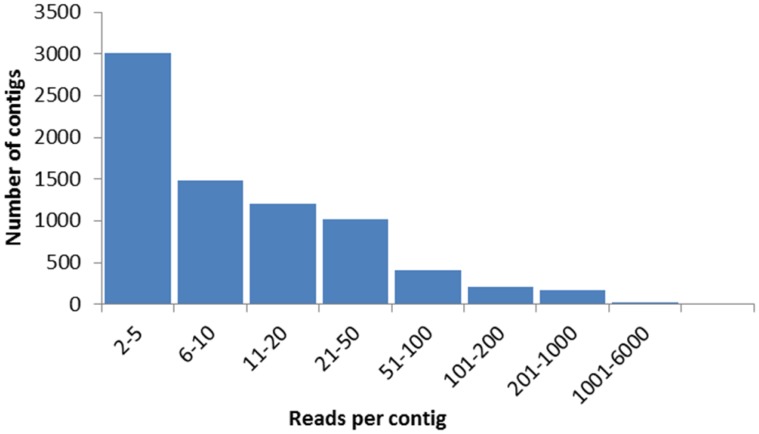
Number of sequences composing the assembled *An. aquasalis* contigs. A total of 7544 contigs were assembled from ≥2 sequences. The number of sequences that compose each contig varies from 2 to 5,207, with an average of 35 sequences per contig. Forty-three percent of the assembled contigs contained 10 or more sequences.

Assembled and annotated sequences are available in Anaquexcel database at http://exon.niaid.nih.gov/transcriptome/An_aquasalis/Anaquexcel.xlsx (a condensed table with basic information was also provided at http://exon.niaid.nih.gov/transcriptome/An_aquasalis/Anoaqua-Summarized.xlsx). A summary of the assembly compared to the raw reads is shown in Figure S1. The Anaquexcel database contains 7544 contigs assembled from ≥2 sequences and 1999 singletons. The number of sequences that compose each contig varies widely (from 2 to 5,207, average of 35 sequences per contig); 43% of the assembled contigs contained 10 or more sequences (Figure 1).

**Figure 2:**
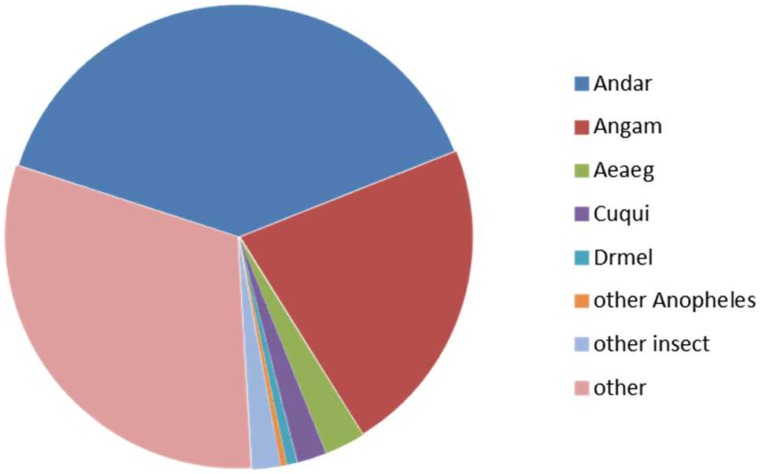
Distribution of the best matches of all *An. aquasalis* predicted proteins by organisms: Andar- *An. darlingi*; Angam- *An. gambiae*; Aeaeg, *Ae. aegypti*; Cuqui, *Cu. quinquefasciatus*; Drmel, *D. melanogaster*; Other *Anopheles* species; Other insects- not of the *Anopheles* genus; Other- non-insect organisms.

**Figure 3:**
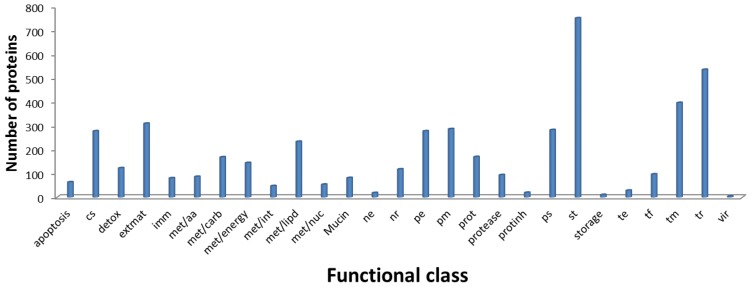
Classification of the annotated *An. aquasalis* proteins in functional categories. The numbers in parenthesis indicate the number of proteins in each category. apoptosis (64), cs- Cytoskeletal (278), detox- Detoxification (123), extmat- Extracellular matrix and adhesion (310), imm- Immunity (81), met/aa- Amino acids metabolism (87), met/carb- Carbohydrate metabolism (169), met/energy- Energy metabolism (145), met/int- Intermediary metabolism (48), met/lipd- Lipid metabolism (234), met/nuc- Nucleotide metabolism (54), mucin (82), ne- Nuclear export (19), nr- Nuclear regulation (118), pe- Protein export machinery (278), pm- Protein modification machinery (287), prot- Proteasome machinery (170), protease (94), protinh- Protease inhibitor (20), ps- Protein synthesis (283), st- Signal transduction (752), storage (11), te- Transposable element (29), tf- Transcription factor (97), tm- Transcription machinery (397), tr- Transporters and storage (536), vir- Viral product (4).

Blastp analyses of the deduced *An. aquasalis* protein sequences indicated that 70% of them have their closest counterpart in another insect (39% *An. darlingi*; 22% *An. gambiae*; 3% *Ae. aegypti*; 2% *Cu. quinquefasciatus*; and 4% other insects) (Figure 2). The percentage coverage was at least 50% for 4,671 proteins and 2,057 had coverage of at least 90%. Furthermore, 6,495 of the blastx searches resulted in best hits with at least 50% identity. These results support the proposed functional annotation of the majority of the deduced *An. aquasalis* proteins (Figure 3). The translated products of 4,821 contigs or singletons were not assigned a putative function (classified as unknown) either because efforts failed to identify similar products in all the searched databases or because similar proteins identified in other organisms have no assigned biological role or activity. Among those with unknown functions, 1,434 encode products similar to proteins found in other organisms (conserved hypothetical proteins). The remaining 2,387 transcripts classified as having unknown functions either encode novel proteins or alternatively correspond to fragments from mRNAs untranslated regions (UTRs) or non-protein encoding RNAs. The predicted presence of amino terminal secretory signal peptide-like sequences supports the conclusion that 15% of the translation products are putatively secreted proteins. These data complement and extend the analyses of EST databases derived from *An. gambiae* mosquitoes in similar physiological conditions [40]–[44].

To better detail the data generated by RNA-seq, we elaborated 7 sections below to discuss results related to larva and adult enhanced and specific transcripts, the adult female *An. aquasalis* sialome, blood meal regulated transcripts, immunity-related transcripts, signaling networks in *An. aquasalis* and conservation of gene regulation between *An. aquasalis* and *An. gambiae*. In order to support our data, we validated the expression levels of 12 contigs by qRT-PCR experiments, being 5 contigs chosen from blood meal regulated transcripts, 5 chosen from larva enhanced and specific transcripts and 2 chosen from adult enhanced and specific transcripts (Supplementary data, Figure S2, S3 and S4).

### Larva enhanced and specific transcripts

One hundred twenty one transcripts were represented by at least 10 reads in the larval sample and were not detected in adults. Transcripts encoding hexamerins were among those accumulated most in larvae (Table S1 and S2). This finding is consistent with previous descriptions of hexamerins, also referred to as larval serum proteins (LSP) or insect storage proteins, as abundant proteins at the late larval stages of holometabolous insects [45], [46]. Hexamerins are synthesized in the larval fat body, secreted into the hemolymph and taken up by fat body shortly before pupation. These proteins degrade during metamorphosis providing a source of amino acids for energy production and adult protein synthesis. Both LSP-1 like (contigs 1020, 1221, 1222, 1226, 3211 and 23555) and LSP-2 like (contigs 637, 2009 and 7437) hexamerins were found. Hexamerin-encoding transcripts corresponding to contig 1221 were found highly accumulated in both larvae and adults. Hexamerins expressed in adult mosquitoes have been reported [47] but their function during this developmental stage is unknown. Abundant larval mRNAs encoding ribosomal proteins, translation initiation factors and elongation factors represent the active protein synthesis machinery, consistent with the rapid growth rate during the third and fourth instars of mosquito larvae. Several transcripts encoding cuticle proteins have >50 reads in larvae and were not detected in adults (contigs 397, 983, 1598, 4095 and 17360). Developmental stage specific-cuticular protein transcripts were reported in *An. gambiae* [48], and those and our data support the hypothesis that the stage-specific expressed proteins are components of the different cuticular structures that characterize each metamorphic stage.

### Adult enhanced and specific transcripts

A total of 424 transcripts represented by at least 10 reads in the adult dataset were not found in larvae. Transcripts enhanced in non-blood fed adults include components of visual sensory organs (Table S1 and S2). Opsins and arrestins (contigs 618, 1471 and 1843) are abundant components of the adult insect compound eyes and expressed highly in adult insects. Arrestins are important components for desensitization of G protein-coupled receptor cascades that mediate neurotransmission as well as olfactory and visual sensory reception [49], [50]. Other components of the olfactory system with enhanced or specific expression in adults include16 identified contigs encoding odorant binding proteins, 10 of which were not represented in the sequenced larval RNA sample. These latter are indicative of new functions in the olfactory system that are specific to adult mosquitoes, such as host finding and breeding site selection.

Structural and enzymatic components of the digestive system also were among the adult specific transcripts. An adult-specific peritrophin (contig 1702) component of the peritrophic matrix (PM) was identified. A number of functions have been attributed to the PM, including protection against pathogens and abrasion, and compartmentalization of digestion [51], [52]. The PM may delay digestion in adult mosquitoes [53] and modulate malaria parasites development [54]. Digestive trypsins with an expression pattern similar to those of *An. gambiae* trypsins 3, 4 and 7 were detected. These trypsins (contigs 457,1977 and 8935) are expressed exclusively in adults and are down-regulated following a blood meal [55]. These trypsin-like enzymes are probably necessary at the initial steps of digestion, but are dispensable later. Alternatively, their functions may be unrelated to digestion and they could associate with other processes regulated by limited proteolysis of precursors. Precursor proteins often require processing at specific sites in order to release their bioactive products [56], [57]. Contig 383 corresponds to a transcript encoding an adult specific cuticle protein further supporting the hypothesis that the stage-specific cuticular proteins make up the different cuticular structures of larvae and adult insects [48].

### The adult female *An. aquasalis* sialome

Although the *An. aquasalis* transcriptome presented here was performed with whole body-extracted RNA and the salivary glands are only a small percentage of the total tissue, possibly less than 0.1%, (generally containing 1–3 µg protein), several putative salivary proteins of the adult female *An. aquasalis* were identified, based on their similarity to a database of salivary proteins from blood feeding Nematocera [58]. We considered only transcripts that are significantly up-regulated in the adult libraries as compared to the larval library as indicated in the methods section. Among putative salivary enzymes, contigs 5562, 17508 and 8234 encode members of the 5′nucleotidase/apyrase families, which are inhibitors of platelet aggregation. These contigs were assembled from 22–62 reads from adult libraries and 0–1 from larvae; similarly, contig 9665 codes for an alkaline phosphatase 66% identical to the *Aedes aegypti* salivary enzyme and was assembled from 60 reads from adults but zero from larvae. Although many serine peptidases were found in the *An. aquasalis* transcriptome, those encoded by contigs 2013 and 20556 are most similar to previously-described mosquito salivary enzymes that may play a role in blood feeding, such as destroying fibrin clots. Three peroxiredoxins similar to previously-described salivary proteins are also enriched in the adult libraries (contigs 4115, 19752 and 2971). Maltases have been described in mosquito salivary glands and are found in both male and female glands and assist sugar feeding. Contig 10431 with 45 reads has no larval reads and is a candidate for encoding a salivary enzyme.

Mosquito sialomes also contain antimicrobial and immune components found in both male and females including pathogen-recognition proteins and classical antimicrobial peptides. The antimicrobial peptide gambicin (contig 120, 70 reads from adults, 2 from larvae, 8.4 relative fold enrichment in adult library), three chitinase-like proteins (contigs 568, 3554 and 3923), a protein with an ML domain involved in pathogen lipid recognition (contig 4200) and a GGY family peptide (contig 10227) are possible salivary enriched gene products. The antigen-5 family of proteins is found ubiquitously in animal and plants, and specific family members are expressed in virtually all sialomes studied so far. Contig 436 (76 reads from adults and zero from larvae) is most similar to previously-annotated salivary members of this family.

A more specific set of proteins found only in mosquito or Nematoceran sialomes also were discovered. This group includes four members of the Aegyptin family of inhibitors of collagen-induced platelet aggregation (contigs 2621, 5929, 5148 and 1583) assembled from 843 adult reads but only three from the larval library. Members of the D7 family, involved in binding of host biogenic amines and inflammatory prostanoids also were assembled solely from adult-derived reads (contigs 2060, 950, 4474 and 19124). The *Anopheles*-specific antithrombin, anophelin, was matched in contig 801, assembled from 139 and 5 adult and larval reads, respectively. The uniquely anopheline salivary protein family, SG1/Trio, with unknown function, is represented by contigs 148, 3403, 21751, 2539 and 1630, assembled from 410 and 21 adult and larval reads, respectively. Further studies demonstrating the salivary specificity of these transcripts are needed.

### Blood meal regulated transcripts

Fifty one transcripts not detected in larvae and sugar-fed mosquito samples were represented by ≥10 reads in the RNA samples of blood-fed females (Table S1 and S2).

*Blood Digestion* - The up-regulation of enzymes and proteins involved in digestion following a blood meal is well-documented in mosquitoes [55], [59]–[61], including *An. aquasalis* [22], [23], [62]. Accordingly, the transcription products of several serine peptidases (contigs 299 and 305, AnaqTryp-1 and -2, respectively; contigs 2013 and 10625, Anachy1 and Anachy2 respectively), aminopeptidases and carboxypeptidases (contigs 2537 and 31670) were identified in our study as increasing in abundance following blood feeding.

Mucins, peritrophins (contigs 5059 and 9948) and G12 microvillar proteins (contig 6859) are up-regulated following blood meal in *An. aquasalis* and other insects. The accumulation of these proteins in the mosquito midgut lumen at a time when malaria parasites are traversing the midgut epithelium has been linked to a possible role in modulation of malaria parasites development [54], [63]–[65].

*Oogenesis* - Genes encoding vitellogenins, other yolk components and eggshell-related proteins (vitellogenins, contigs 407 and 408; vitellogenic cathepsin, contig 1783) were identified as having large increases in transcript abundance after a blood meal. Our data confirm previously-described increase in vitellogenic protein expression in blood-fed mosquitoes [66]. Contig 24500 encodes lipophorin, a lipid transporter crucial for oogenesis, up-regulated following blood meal in *An. aquasalis* as well as other mosquitoes [43]. In addition to their indispensable function in mosquito reproduction, lipophorin and vitellogenin reduce parasite-killing by the antiparasitic factor TEP1 [67]. In the absence of either one, TEP1 binding to the ookinete surface becomes more efficient.

Eggshell proteins are expressed abundantly in the ovaries of vitellogenic insects. These proteins and related gene products have been studied extensively in model organisms such as *Drosophila melanogaster* and *Bombyx mori* [68], and more recently in *An. gambiae* [69]. We identified up-regulated transcripts encoding structural (vitelline membrane proteins, contigs 296, 2546 and 2709) as well enzymatic components of the mosquito eggshell (chorion peroxidase, contig 3237). Several other transcripts associated with egg formation and embryo development also were included in the group of blood meal induced transcripts (for example, Maternal protein exuperantia, contig 1219; Tyrosine-protein kinase receptor torso, contig 19837).

Transgenic mosquito strains are being developed to contribute to the control of malaria transmission [70]. The genetics and resulting phenotypes of a female-specific RIDL strategy, previously developed for dengue vector mosquitoes [71] has been adapted to a vector of human malaria, *An. stephensi* [72], indicating that the approach is applicable to other anopheline species. Moreover, production of malaria parasites-resistant transgenic mosquitoes has also been achieved in several laboratories [73]. The translation of these new, genetics-based technologies to new world anopheline species is likely feasible and the successful colonization of *An. aquasalis* places these mosquitoes in the list of target species for transgenesis. Therefore, regulatory sequences that drive transgenes expression in the appropriate developmental stage and organ, and produce an optimum amount of product are required [74], [75] and the blood meal-activated genes identified in our work provide candidates for isolation and characterization of *An. aquasalis* functional promoters.

One hundred and ninety transcripts decrease significantly in abundance after the blood meal, most of which encode proteins with unknown functions. These likely represent genes expressed in tissues that are not involved in digestive and oogenesis functions. Alternatively, these genes could encode products such as early trypsins (contig 989) necessary at the initial steps of the digestion, but dispensable later [76], [77].

### Immunity-related transcripts

The mosquito immune system plays a critical role in limiting the spread of malaria and other vector-borne diseases. We identified a series of components of the innate immune system of *An. aquasalis*, including the antimicrobial peptides defensin (contigs 30436, 21901 and 1968), attacin (contig 20438), cecropin (contig 120), gambicin (contig 3403) lysozyme-c (contigs 4061, 1957, 14486, 16360 and 13712) and lysozyme-i (contigs 28407 and 27701). Additionally, members of the Toll pathway (Toll, contig 15063, cactus, contig 1543, dorsal, contig 17521, Kenny, contig 11522), members of the IMD pathway (IMD) (DIAP2, contig 6046, IKKbeta, contig 10934) and thioester proteins (TEPs) (contigs 21929, 29052 and 32589), the three major immune response systems in dipterans insects were identified.

Recent research supported differences between the responses of *An. aquasalis* to *P. vivax* infection when compared to immune response of *An. gambiae* to *P. falciparum* [18], [19], [78]. The immunity-related transcripts identified in this study will allow a more detailed study of the immune response of this neotropical vector to both *P. vivax* and *P. falciparum* infection.

### Signaling networks in *An. aquasalis*

The dynamics of tyrosine phosphorylation-dephosphorylation constitutes a master biochemical regulator of cell biology [79]. It is mediated by a set of three major components: protein tyrosine kinases (PTKs), protein tyrosine phosphatases (PTPs), and Src Homology 2 (SH2) domains. It has been demonstrated previously the role of such a mechanism during tick embryogenesis [80], mosquito early adult development [81] and parasite infection [82]. The analysis of the *An. aquasalis* tyrosine phosphorylation-dephosphorylation regulatory enzymes revealed two contigs encoding PTPs (contigs 10198 and 21444). The first, contig 10198, encodes the classical non-receptor PTP (PTPn9) and the number of its reads increases in the larval/adult transition but decreases after blood feeding. This is a soluble tyrosine phosphatase that down-regulates prolactin- and EGF-mediated STAT5 activation [83]. STAT5 regulates expression of genes that promote cell survival and proliferation in breast cancer cells. The levels of phosphorylated EGF also are increased upon the suppression of PTPn9 mediated by MicroRNA miR-24 [84].

The *Anopheles* family of STAT transcription factors (Ag-STAT) was reported to be activated by bacterial challenge which then results in their nuclear translocation. This pathway is activated by inhibitors of PTPs [85]. We speculate that when bacterial loads increase following a blood meal, the suppression of PTPn9 expression allows the establishment of a STAT-mediated immunity and the induction of cell growth by incoming nutrients derived from blood digestion.

The second transcript, contig 21444, encodes for a dual-specificity phosphatase (DUSP), a PTP that also increases during the larval to adult transition and is strongly suppressed after blood feeding. These enzymes dephosphorylate both phosphotyrosine and phosphothreonine residues in target proteins and act as deactivators of mitogen-activated PK (MAPK) cascades. The complete set of genes in *An. gambiae* encoding for MAPKs and their activation profiles were described [86]. The level of phosphorylation of MAPKs in *Anopheles* was demonstrated to be responsive through treatment with insulin, TGF-B1, and LPS [86]. Curiously, p38 phosphorylation also is affected by hydrogen peroxide treatment, a common inhibitor of PTPs. So it is likely that contig 21444 is the enzyme that ultimately down-regulates the level of MAPK activation after cell treatment with the above-mentioned agents. The induction of MAPK activation following both metabolic and immune challenges after blood feeding coincides with the down-regulation of contig 21444 transcripts.

An overall analysis of signaling molecules involved in direct phosphorylation-dephosphorylation circuits whose transcripts are up-regulated during different stages of mosquito development revealed one contig (1237) encoding a CBL-interacting serine/threonine-protein kinase 10 present in larvae (Table S2). Calcineurin B-like proteins (CBLs) are calcium binding proteins that interact in the presence of calcium with a group of serine/threonine kinases designated as CBL-interacting protein kinases (CIPKs). This signaling network in plants allows the coupling of several different types of stress to a specific response. The most common is the regulation of salt stress [87]. Since *An. aquasalis* larvae are highly tolerant to salt stress, the CIPKs could be part of a similar response in which overexpression of these genes promotes salt tolerance [88], [89].

The DUSP (contig 21444) mentioned above and a serine/threonine-protein phosphatase 4 regulatory subunit 1 (PP4) (contig 20076) are among the most down-regulated transcripts following a blood meal (Table S2). Serine/threonine phosphatases are divided in two main families, PPP and PPM. PPP are divided in five subtypes (PP1, PP2A, PP3, PP5 and PP7). PP4 protein belongs to the PP2A subfamily and like this enzyme is modulated by R regulatory subunits [90], [91]. PP4 is likely involved in the repair of DNA double-strand breaks but it was recently demonstrated that it also acts as a negative regulator of negative regulator of NF-κB activity in T lymphocytes [92], [93]. PP4R1 provides the interaction between the IκB kinase (IKK) complex and the phosphatase PP4c, thereby dephosphorylating and inactivating the IKK complex. The inactivation of IKK complex blocks NF-kB activation once its inhibitors, called IκBs (Inhibitor of κB), remain bound to the NF-kB complex. Deficiency of PP4R1 caused sustained and increased IKK activity and thus the permanent inhibition of immune responses [93]. Mosquito Rel1 and Rel2 members of the NF-kB transcription factors are activated after a blood meal and their silencing blocks the establishment of an immune response against malaria parasite. This occurs due to the inhibition of the basal expression of the anti-plasmodium genes TEP1 and LRIM,1 which are involved in the mosquito resistance to malaria parasite [94]. Thus PP4 might represent a long term down regulator of NF-kB activation and its 9-fold down regulation after a blood meal supports the hypothesis that it is required to enhance mosquito refractoriness to eventual pathogen infection. Future molecular analysis of such pathways together with the precise identification of the phosphorylation sites affected may reveal novel targets to overcome disease transmission by anophelines.

### Conservation of gene regulation between *An. aquasalis* and *An. gambiae*

**Figure 4:**
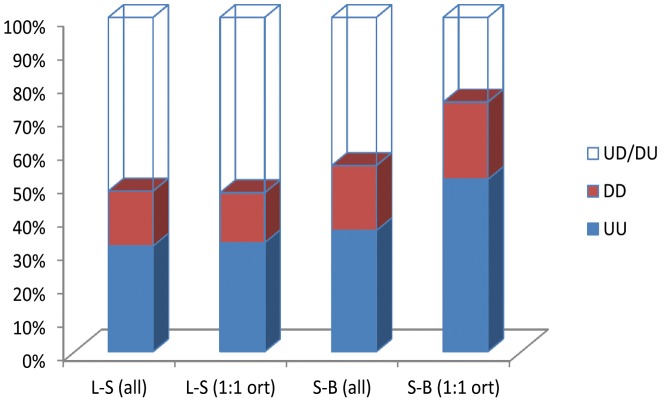
Comparisons of developmental changes in gene expression between *An. aquasalis* and *An. gambiae*. Developmental gene regulations [up(U) or down(D)-regulation] between larvae and sugar fed females (L-S all) or between sugar fed females and blood fed females (S-B all) of *An. aquasalis* transcripts that have a homolog *An. gambiae* (best Blast match) represented in the GeneChip *Plasmodium*/*Anopheles* Genome Array [42], [95] were compared. The pairwise comparisons including all *An. aquasalis*/*An. gambiae* homologous pairs of genes demonstrated a lack of conservation of developmental changes in transcript abundance between the two mosquito species. Similar analyses restricting the transcript list to putative 1∶1 ortholog pairs, defined by reciprocal blast and only those significantly regulated in *An. aquasalis*, with at least 3 fold change between two compared samples (L-S 1∶1 ort or S-B 1∶1 ort) showed that 75% the transcripts regulated by blood feeding were consistently up or down regulated in both species. Using the same restricted list of transcripts, only 49% of the transcripts were consistently up- or down-regulated between L-S in both species. Genes up-regulated or down-regulated in both species are indicated by (UU) or (DD), respectively. Transcripts differentially regulated between the two species are indicated by (UD/DU).

The changes in transcript abundance between larvae and adults and between sugar fed and blood fed females observed in our study were compared with those previously described for *An. gambiae* in similar developmental stages. A total of 8,355 contigs presented here had a homolog *An. gambiae* transcript (best Blast match) represented in the GeneChip *Plasmodium*/*Anopheles* Genome Array [42], [95]. The pairwise comparisons including all *An. aquasalis*/*An. gambiae* homologous pairs demonstrated a lack of conservation of developmental changes in gene expression between the two mosquito species. Approximately half of the genes showed consistent up or down regulation in both species while the remaining showed up regulation in one mosquito and down in the other (Figure 4).

A more stringent analysis was performed by restricting the transcript list to putative 1∶1 orthologous pairs defined by reciprocal blast. Additionally, the list included only those regulated significantly in *An. aquasalis*, and with ≥3-fold change between two compared samples (larvae/sugar fed adult females or sugar fed females/blood fed females). Applying these more stringent parameters, 75% of the transcripts regulated by blood feeding were consistently up- or down-regulated in both species. The same was not observed for the transcripts accumulated differentially between larvae and adults where only 49% were consistently up- or down-regulated in both species (Figure 4).

This study represents the first effort to sequence the transcriptome of the New World malaria vector, *An. aquasalis*. We have explored the transcriptomes of larva and adult *An. aquasalis*, providing valuable information about protein-coding transcripts involved in biological processes relevant to mosquito development, blood feeding, blood digestion, reproduction, and the *Plasmodium* life cycle. This study, together with other recently published and ongoing efforts to sequence the genomes and transcriptomes of malaria vectors (vectorbase.org) [96]–[98], will provide a needed and more complete understanding of malaria vector biology.

Our findings on gene functionalities shed light on the essential physiology of *An. aquasalis* and thus may help one to develop new control strategies. Moreover, present data may act as shortcuts to investigate genes of other congeneric pathogen-vectors. Data may also be used as taxonomic molecular markers or in future phylogenetic inferences (of genes or species) based on exons which are not under differential between-taxa natural selection.

Finally, one limitation of the sequencing project reported here is that transcripts present only at developmental stages not included in this study (embryos, pupae, adult males) could not be detected. It also is important to be aware that accumulation levels and variations in transcript abundance may not correlate with a similar variation in the amount of the encoded protein. Furthermore, enzyme activity may be subject to regulation by feedback inhibition by the corresponding pathway product, allosteric interactions, reversible covalent modifications or programmed proteolytic cleavage.

Differently from other Anophelines, the complete genome sequence of the *An. aquasalis* was not obtained until now. This fact imposes a limitation to estimate how complete this transcriptome is, and the size of coding genome as well as orthology comparison between related species needs to be adopted with this assumption.

Further studies to generate a comprehensive picture of gene expression, protein synthesis and function throughout the mosquito development are needed to uncover biological processes in mosquitoes and to help in the efforts to control malaria transmission.

## Supporting Information

Figure S1 Summary of assembly compared to the raw reads. A. Number of sequences in raw file in relation to sequence size. B. Number of reads in assembled results in relation to sequence size.
(TIF)

Figure S2 Validation of blood meal regulated transcripts by qRT-PCR. Expression levels of 5 genes (megaclu_asbSigP-9948, Anoaqua-3237, megaclu_asbSigP-2537, Anoaqua-5059 and Anoaqua-24500) were determined in samples from *An. aquasalis* L3/L4 larvae pools (Larvae), adult females fed on sucrose (Sucrose-fed) and adult females 24 hs post blood meal (Blood-fed). Relative mRNA levels are displayed as the mean of fold differences in relation to the calibrator sample (Larvae) and error bars represent the standard error of the mean (± S.E.M.). Expression levels of megaclu_asbSigP-9948 in blood-fed samples are significant different in relation to other samples (Kruskal-Wallis test. *, *p*<0.0001). Expression levels of Anoaqua-3237, megaclu_asbSigP-2537, Anoaqua-5059 and Anoaqua-24500 in blood-fed samples are significant different in relation to other measured samples (One way ANOVA test. *, *p*<0.0001).
(TIF)

Figure S3 Validation of larva enhanced and specific transcripts by qRT-PCR. Expression levels of 5 genes (Anoaqua-397, Anoaqua-4095, Anoaqua-1222, Anoaqua-1598 and Anoaqua-17360) were determined in samples from *An. aquasalis* L3/L4 larvae pools (Larvae), adult females fed on sucrose (Sucrose-fed) and adult females 24 hs post blood meal (Blood-fed). Relative mRNA levels are displayed as the mean of fold differences in relation to calibrator sample (Sucrose-fed for Anoaqua-397 and Anoaqua-4095; Blood-fed for Anoaqua-1222, Anoaqua-1598 and Anoaqua-17360) and error bars represent the standard error of the mean (± S.E.M.). Expression levels of Anoaqua-397, Anoaqua-4095, Anoaqua-1222 and Anoaqua-1598 in Larvae samples are significant different in relation to other measured samples (One way ANOVA test. *, *p*<0.0001). Expression levels of Anoaqua-17360 in Larvae samples are significant different in relation to other samples (Kruskal-Wallis test. *, *p*<0.0001).
(TIF)

Figure S4 Validation of adult enhanced and specific transcripts by qRT-PCR. Expression levels of 2 genes (Anoaqua-436 and Anoaqua-457) were determined in samples from *An. aquasalis* L3/L4 larvae pools (Larvae), adult females fed on sucrose (Sucrose-fed) and adult females 24 hs post blood meal (Blood-fed). Relative mRNA levels are displayed as the mean of fold differences in relation to calibrator sample (Larvae) and error bars represent the standard error of the mean (± S.E.M.). Expression levels of Anoaqua-436 and Anoaqua-457 in Larvae samples are significant different in relation to other measured samples (One way ANOVA test, *, *p*<0.0001).
(TIF)

Table S1 Most abundant transcripts in larvae, sugar fed and blood fed adult female *An. aquasalis*.
(XLSX)

Table S2 Most up or down-regulated transcripts during *An.aquasalis* development and following a blood meal.
(XLSX)

Table S3 Primer pairs used for qRT-PCR validation and PCR efficiency of the targets
(XLSX)

Table S4 Validation of Rp49 as housekeeping gene to normalize expression values of selected candidates.
(XLSX)
