# Supplementary figures and images for: Transcriptome Sequencing and Developmental Regulation of Gene Expression in Anopheles aquasalis
Source: PLoS Negl Trop Dis. 2014 Jul 17;8(7):e3005. doi: 10.1371/journal.pntd.0003005 (PMC4102416; doi:10.1371/journal.pntd.0003005)

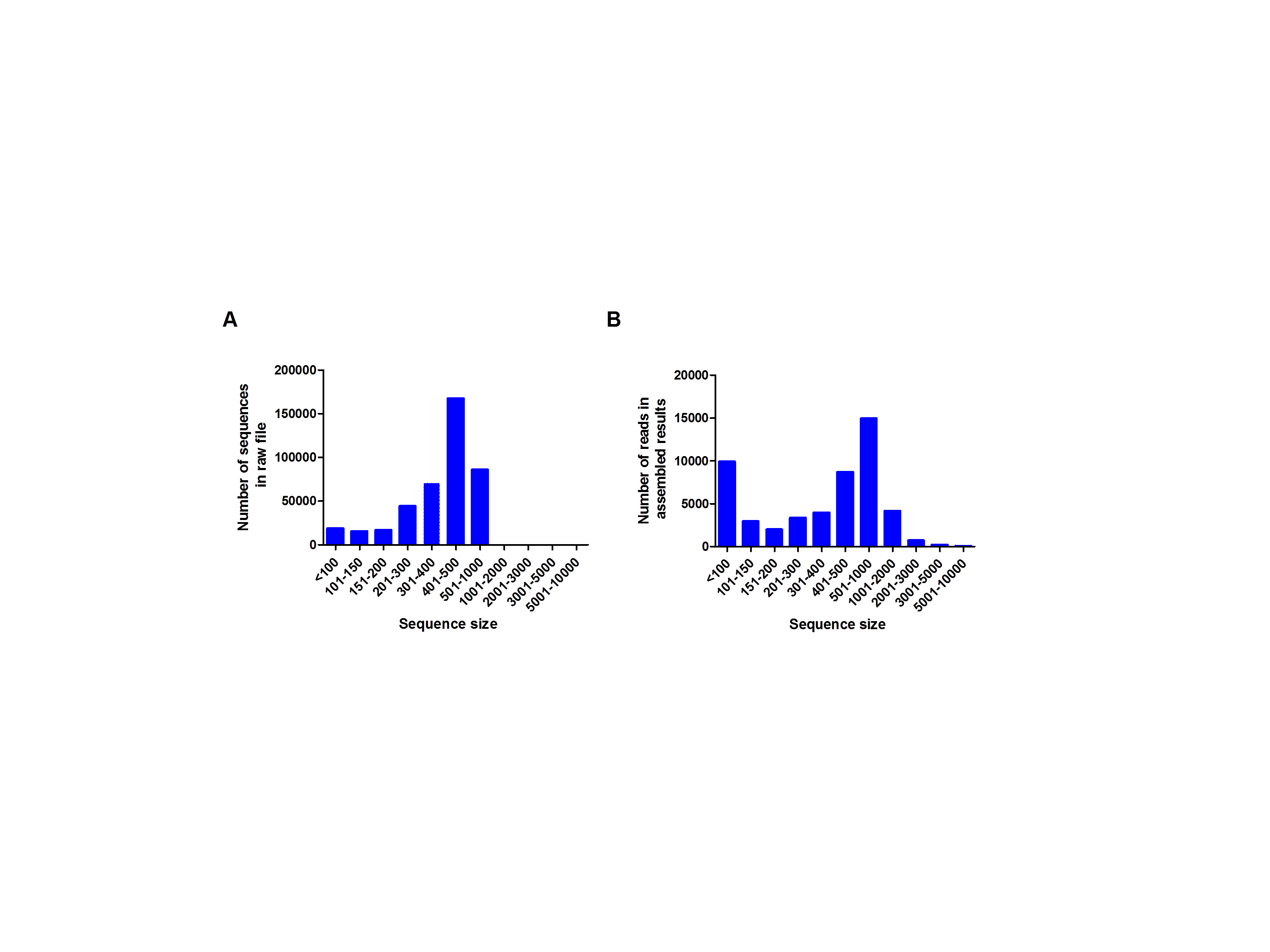

Supplement: Figure S1 — Summary of assembly compared to the raw reads. A. Number of sequences in raw file in relation to sequence size. B. Number of reads in assembled results in relation to sequence size. (TIF) [file pntd.0003005.s001.tif]

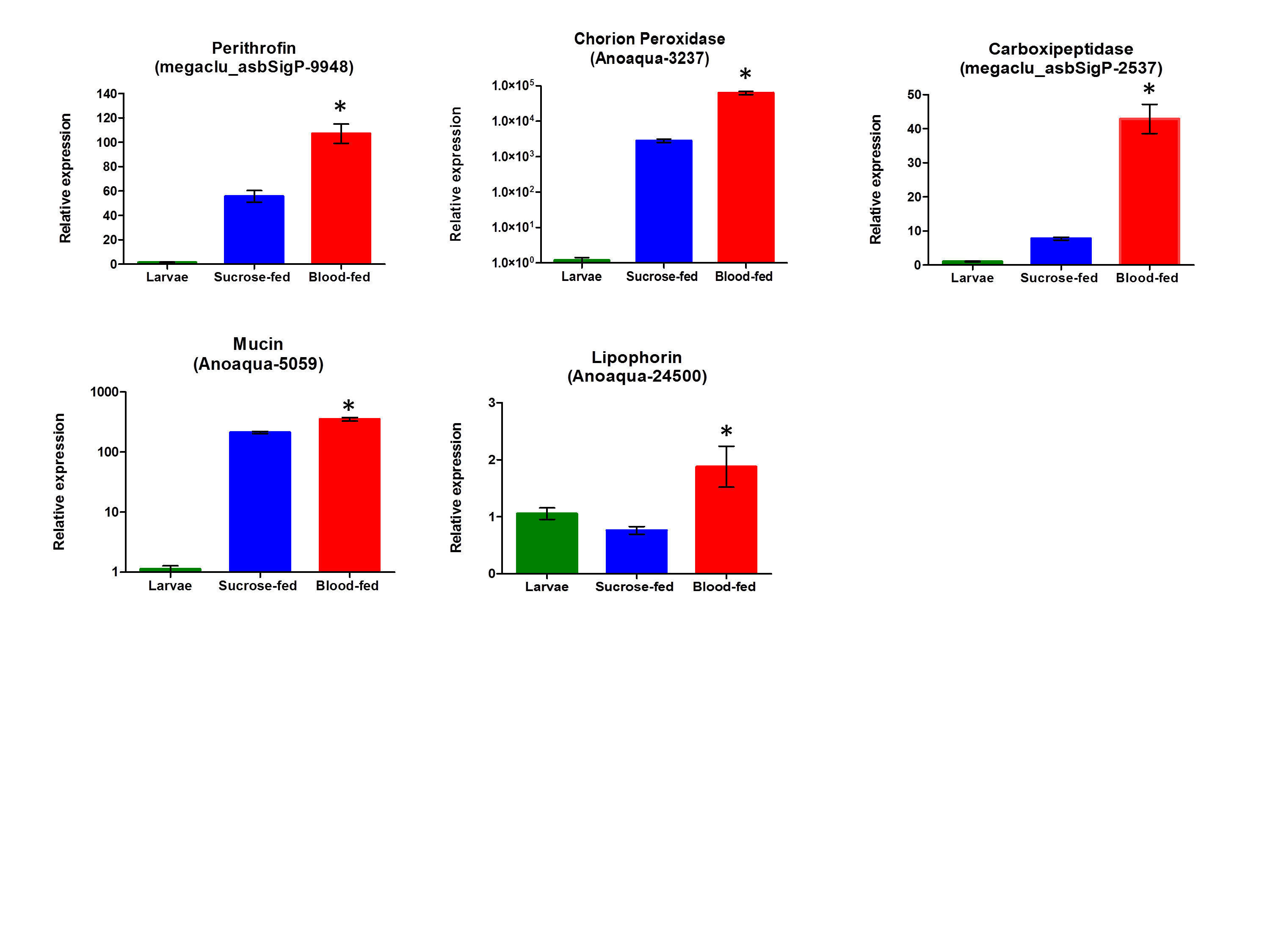

Supplement: Figure S2 — Validation of blood meal regulated transcripts by qRT-PCR. Expression levels of 5 genes (megaclu_asbSigP-9948, Anoaqua-3237, megaclu_asbSigP-2537, Anoaqua-5059 and Anoaqua-24500) were determined in samples from An. aquasalis L3/L4 larvae pools (Larvae), adult females fed on sucrose (Sucrose-fed) and adult females 24 hs post blood meal (Blood-fed). Relative mRNA levels are displayed as the mean of fold differences in relation to the calibrator sample (Larvae) and error bars represent the standard error of the mean (± S.E.M.). Expression levels of megaclu_asbSigP-9948 in blood-fed samples are significant different in relation to other samples (Kruskal-Wallis test. *, p<0.0001). Expression levels of Anoaqua-3237, megaclu_asbSigP-2537, Anoaqua-5059 and Anoaqua-24500 in blood-fed samples are significant different in relation to other measured samples (One way ANOVA test. *, p<0.0001). (TIF) [file pntd.0003005.s002.tif]

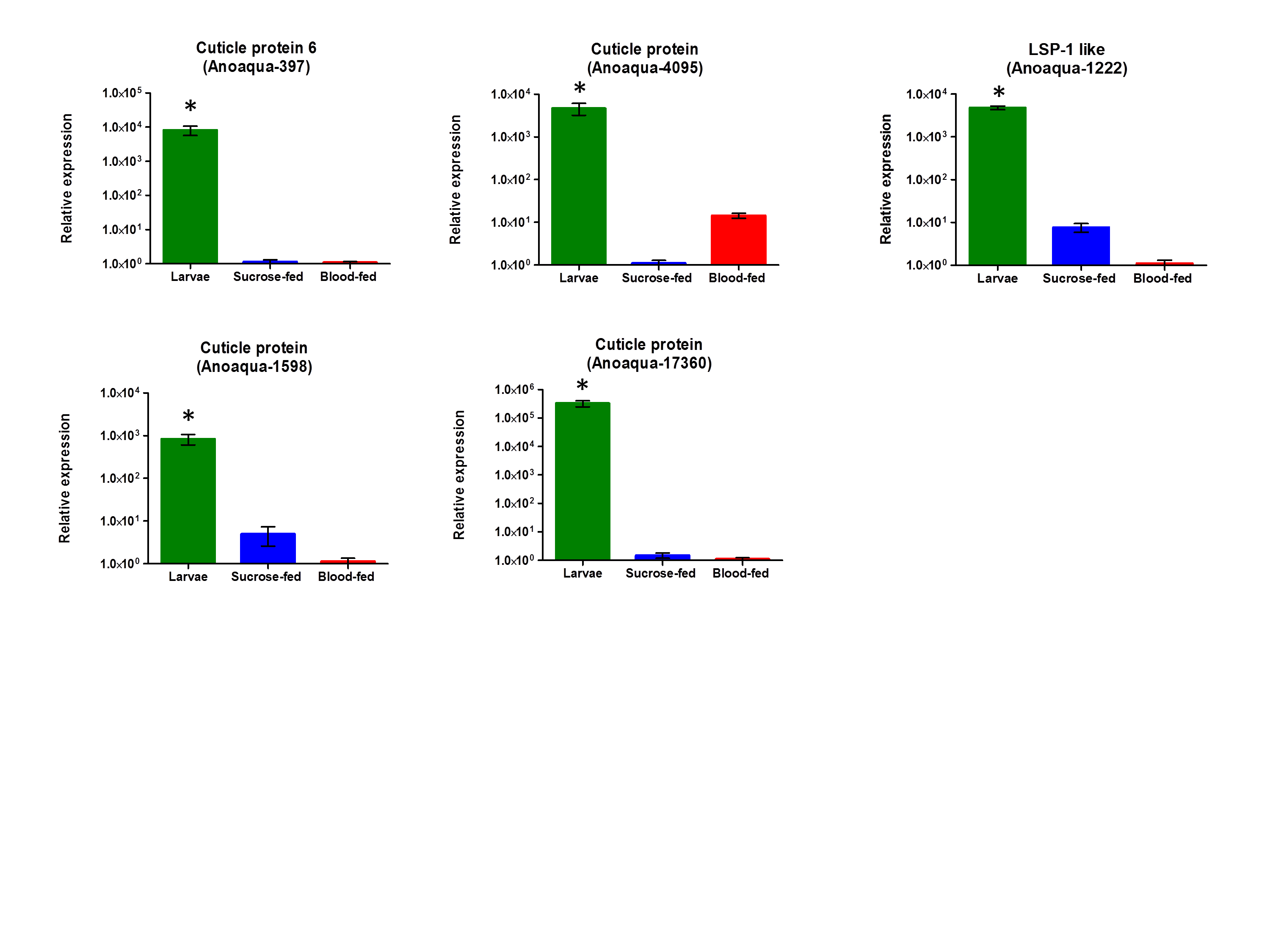

Supplement: Figure S3 — Validation of larva enhanced and specific transcripts by qRT-PCR. Expression levels of 5 genes (Anoaqua-397, Anoaqua-4095, Anoaqua-1222, Anoaqua-1598 and Anoaqua-17360) were determined in samples from An. aquasalis L3/L4 larvae pools (Larvae), adult females fed on sucrose (Sucrose-fed) and adult females 24 hs post blood meal (Blood-fed). Relative mRNA levels are displayed as the mean of fold differences in relation to calibrator sample (Sucrose-fed for Anoaqua-397 and Anoaqua-4095; Blood-fed for Anoaqua-1222, Anoaqua-1598 and Anoaqua-17360) and error bars represent the standard error of the mean (± S.E.M.). Expression levels of Anoaqua-397, Anoaqua-4095, Anoaqua-1222 and Anoaqua-1598 in Larvae samples are significant different in relation to other measured samples (One way ANOVA test. *, p<0.0001). Expression levels of Anoaqua-17360 in Larvae samples are significant different in relation to other samples (Kruskal-Wallis test. *, p<0.0001). (TIF) [file pntd.0003005.s003.tif]

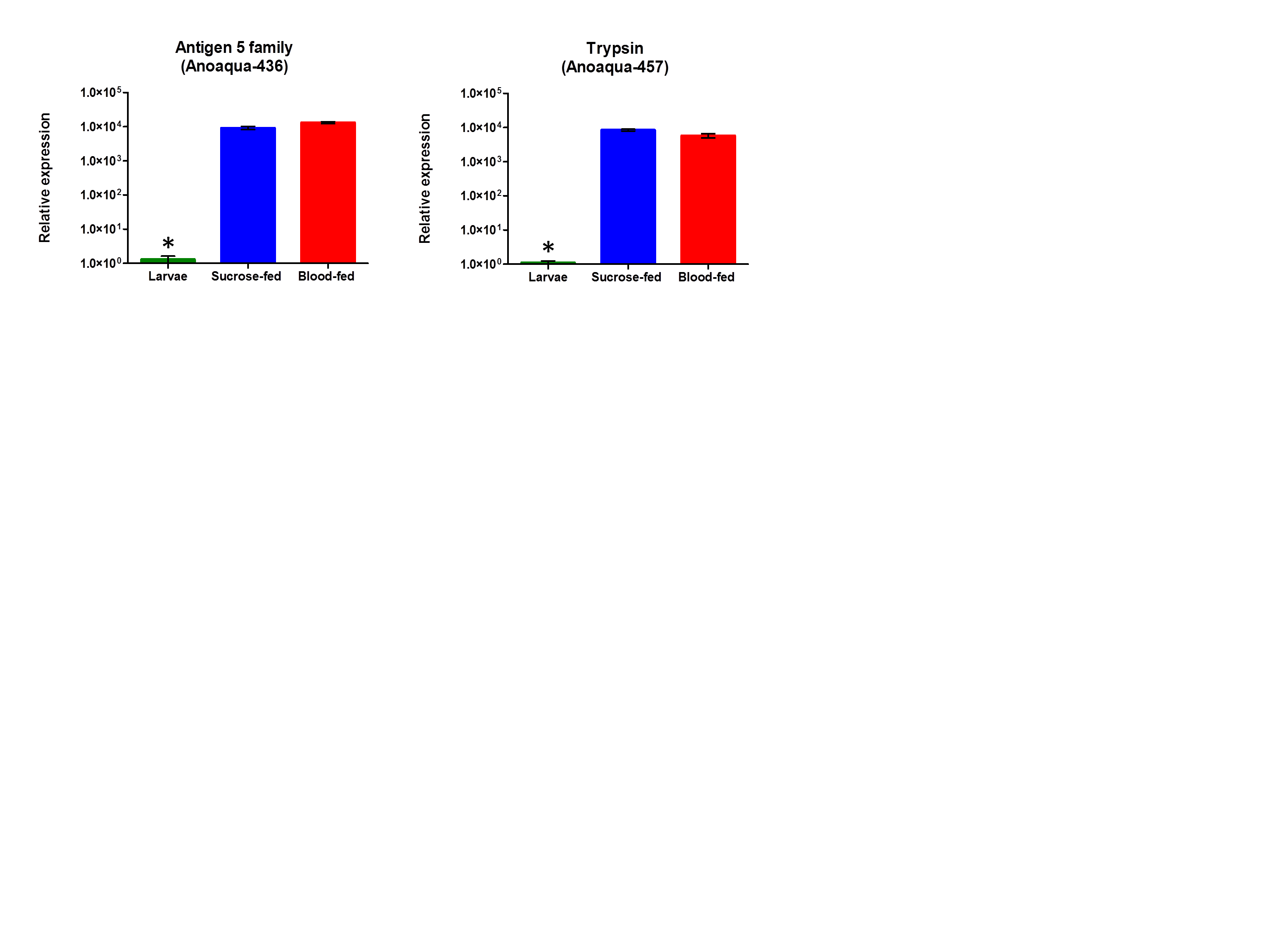

Supplement: Figure S4 — Validation of adult enhanced and specific transcripts by qRT-PCR. Expression levels of 2 genes (Anoaqua-436 and Anoaqua-457) were determined in samples from An. aquasalis L3/L4 larvae pools (Larvae), adult females fed on sucrose (Sucrose-fed) and adult females 24 hs post blood meal (Blood-fed). Relative mRNA levels are displayed as the mean of fold differences in relation to calibrator sample (Larvae) and error bars represent the standard error of the mean (± S.E.M.). Expression levels of Anoaqua-436 and Anoaqua-457 in Larvae samples are significant different in relation to other measured samples (One way ANOVA test, *, p<0.0001). (TIF) [file pntd.0003005.s004.tif]
